# Benzo[a]pyrene activates interleukin-6 induction and suppresses nitric oxide-induced apoptosis in rat vascular smooth muscle cells

**DOI:** 10.1371/journal.pone.0178063

**Published:** 2017-05-22

**Authors:** Huei-Ping Tzeng, Kuo-Cheng Lan, Ting-Hua Yang, Min-Ni Chung, Shing Hwa Liu

**Affiliations:** 1 Institute of Toxicology, College of Medicine, National Taiwan University, Taipei, Taiwan; 2 Department of Emergency Medicine, Tri-Service General Hospital, National Defense Medical Center, Taipei, Taiwan; 3 Department of Otolaryngology, National Taiwan University College of Medicine and National Taiwan University Hospital, Taipei, Taiwan; 4 Department of Pediatrics, College of Medicine, National Taiwan University, Taipei, Taiwan; 5 Department of Medical Research, China Medical University Hospital, China Medical University, Taichung, Taiwan; Max Delbruck Centrum fur Molekulare Medizin Berlin Buch, GERMANY

## Abstract

Benzo[a]pyrene, a ubiquitous environmental pollutant, has been suggested to be capable of initiating and/or accelerating atherosclerosis. Accumulation of vascular smooth muscle cells (VSMCs) in vessel intima is a hallmark of atherosclerosis. Nitric oxide (NO) can suppress VSMCs proliferation and induce VSMCs apoptosis. NO plays a compensatory role in the vascular lesions to reduce proliferation and/or accelerate apoptosis of VSMCs. The aim of this study was to investigate whether benzo[a]pyrene can affect VSMCs growth and apoptosis induced by NO. Benzo[a]pyrene (1–30 μmol/L) did not affect the cell number and cell cycle distribution in VSMCs under serum deprivation condition. Sodium nitroprusside (SNP), a NO donor, decreased cell viability and induced apoptosis in VSMCs. Benzo[a]pyrene significantly suppressed SNP-induced cell viability reduction and apoptosis. VSMCs cultured in conditioned medium from cells treated with benzo[a]pyrene could also prevent SNP-induced apoptosis. Benzo[a]pyrene was capable of inducing the activation of nuclear factor (NF)-κB and phosphorylation of p38 mitogen-activated protein kinase (MAPK) in VSMCs. Both NF-κB inhibitor and p38 MAPK inhibitor significantly reversed the anti-apoptotic effect of benzo[a]pyrene on SNP-treated VSMCs. Incubation of VSMCs with benzo[a]pyrene significantly and dose-dependently increased interleukin (IL)-6 production. A neutralizing antibody to IL-6 effectively reversed the anti-apoptotic effect of benzo[a]pyrene on SNP-treated VSMCs. Taken together, these results demonstrate for the first time that benzo[a]pyrene activates IL-6 induction and protects VSMCs from NO-induced apoptosis. These findings propose a new mechanism for the atherogenic effect of benzo[a]pyrene.

## Introduction

Vascular smooth muscle cells (VSMCs) are responsible for the structural characteristics of the vessel wall, which is important in development, growth, remodeling and repair [1,2]. Many vascular diseases including hypertension, atherosclerosis, post-angioplasty restenosis, and transplant arteriosclerosis are characterized by abnormal VSMCs proliferation and migration, causing VSMCs accumulation in the intima during vascular remodeling [2,3]. Vascular structure and remodeling have been suggested to be determined in large part by a balance between cell growth and cell death by apoptosis [4].

Epidemiological and experimental studies have shown that polycyclic aromatic hydrocarbons (PAHs) are associated with the progression of cardiovascular diseases, including atherosclerosis [5,6]. Benzo[a]pyrene, a major environmental pollutant and a PAH present in tobacco smoke, has been demonstrated to possess the potential of atherogenesis in experimental models [7–10]. The most of studies defining the pathology of benzo[a]pyrene in vascular disease have majorly focused on the abnormal regulation of cell growth/proliferation. However, in addition to changes in the regulation of cell growth, the regulation of cell death by apoptosis may be another important determinant of vessel structure and lesion formation.

Nitric oxide (NO), generated from L-arginine by nitric oxide synthase (NOS), plays diverse physiological functions, such as vascular tonus regulation, neurotransmission, and cytotoxicity [11,12]. NO can exert proapoptotic or anti-apoptotic effects for various cell types [13,14]. Low concentrations of NO (pmol/L-nmol/L) seem to favor cell proliferation/anti-apoptosis and higher concentrations of NO (μmol/L-mmol/L) favor cell cycle arrest or apoptosis in cardiovascular-related cells [13]. NO donors have been shown to affect the cell cycle and suppress proliferation in the aortic VSMCs [15]. In the blood vessels, it has been reported that NO induces apoptosis in vascular endothelial cells [16] and smooth muscle cells [17]. It has also been shown that apoptosis occurs during the process of vascular remodeling and lesion formation [13,18,19]. *In vivo* gene transfer of endothelial NOS resulted in a marked reduction of neointimal formation after balloon injury in rats by constitutively generation of endogenous NO [20]. Furthermore, it has been shown that expression of inducible NOS (iNOS) mRNA and protein is localized not only to macrophages and foam cells but also to VSMCs in atherosclerotic lesions and neointima after balloon angioplasty [21,22]. The iNOS-dependent NO production has been found to act as a survival signal in benzo[a]pyrene-treated rat hepatic epithelial F258 cells via an AhR-regulatory pathway [23]. These observations suggested that NOS expression in the vascular lesions might represent a compensatory mechanism to reduce proliferation and/or accelerate apoptosis of VSMCs through excess generation of NO.

Interleukin-6 (IL-6) is a pleiotropic cytokine. Several studies indicated that IL-6 has critical pathophysiological roles in cardiovascular diseases, such as atherosclerosis [24, 25] and congestive heart failure [26]. Nevertheless, it has been suggested that locally secreted IL-6 is involved in the VSMCs proliferation in response to platelet-derived growth factor (PDGF) [27]. IL-6 could also participate in the 15(S)-hydroxyeicosatetraenoic acid-induced VSMCs migration and neointima formation [28]. IL-6 has also been found to decrease the endothelial NOS activity in human vascular endothelial cells [29]. Moreover, benzo[a]pyrene at a concentration of 10 μmol/L was capable of stimulating the IL-6 secretion in human sebocytes via an AhR signaling pathway [30].

In this study, we hypothesized that benzo[a]pyrene possesses antagonistic potential against NO-related VSMCs death/apoptosis. We investigated the antagonistic effect of benzo[a]pyrene on NO donor-triggered death/apoptosis in a primary rat VSMCs culture model. We also determined whether IL-6 would be a survival mediator in the anti-cell death/apoptotic effect of benzo[a]pyrene on NO donor-treated VSMCs.

## Materials and methods

The protocol for animal study was approved by the Institutional Animal Care and Use Committee, National Taiwan University, College of Medicine, Taipei, Taiwan.

### Primary culture of vascular smooth muscle cells

VSMCs were isolated from the thoracic aortas of male Wistar rats (150–200 g) by the method described previously [31]. Wistar rats were purchased from BioLASCO (Taipei, Taiwan). The study was conducted in accordance with the guidelines of the Animal Research Committee of National Taiwan University, College of Medicine, for the care and use of laboratory animals. Before experiments began, rats were allowed at least 1 week acclimation period at animal quarters with air conditioning and constant humidity. The light was controlled automatically at an interval of 12 h per day. The animals were allowed free access to food and water. To prepare VSMCs, the thoracic aortas were cleaned of fat and adventitia, cut into small strips, and then digested with 1 mg/mL collagenase (Sigma) and 0.125 mg/mL elastase (Sigma) at 37°C for 60 min. Cells were cultured in DMEM containing 10% FCS at 37°C in a humidified atmosphere of 5% CO_2_/95% air. Cells exhibited characteristics of VSMCs were used between the third and sixth passages.

### Analysis of cell number

Cells were seeded at 2×10^4^ cells/well into 12-well plates and allowed to attach overnight. Cells were cultured in serum-free DMEM for 48 h, and then test compounds were added to medium for another 24 h. Cells were harvested, and a 50 μL aliquot was mixed with 0.04% trypan blue and counted twice on a hemocytometer.

### Cell treatment and preparation of total cell lysates

Cells seeded in 6-well plates and grown to 60% to 80% confluence were serum-deprived in DMEM containing 0.1% bovine serum albumin (BSA) for 24–48 h, and treated with or without benzo[a]pyrene, in the presence or absence of sodium nitroprusside (SNP) for indicated time intervals. Cells were then harvested by scraping in 200 μL of ice-cold extraction buffer [50 mmol/L Tris-HCl (pH 7.4), 150 mmol/L NaCl, 10 mmol/L EDTA, 0.1% NP-40, 1 mmol/L orthovanadate, 1 mmol/L PMSF, 10 mmol/L sodium fluoride, 10 μg/mL leupeptin, and 10 μg/mL aprotinin], rotated for 15 min at 4°C, and centrifuged at 10000 × g for 20 min. The supernatant were collected, and stored at -80°C until use.

### Cell cytotoxicity assay

The cytotoxicity was determined using the MTT [3-(4,5-dimethylthiazol-2-yl)-2,5-diphenyltetrazolium bromide] assay (Sigma). Briefly, cells (1×10^4^) were seeded into 96-well plates overnight and starved for 48 h. Then, the medium was aspirated and cells were cultured in serum-free DMEM with vehicle or various concentrations of benzo[a]pyrene solubilized in DMSO in the presence or absence of SNP for indicated time intervals. Subsequently the medium was removed and cells were incubated in medium with MTT (5 μg/mL) for 1 h at 37°C, which was metabolized to formazan, and then dissolved in DMSO and measured in an ELISA.

### Detection of subdiploid DNA population

Cells were harvested and prepared single cell suspension in PBS at 1–2 x 10^6^ cells/mL. Aliquoted 1 mL cells in a 15 mL polypropylene, V-bottomed tube and added 3 mL cold absolute ethanol forcibly in order to prevent clumping and cell loss. Cells were fixed for at least 1 h at 4°C. Cells were washed 2 times with PBS and added 1 mL of 50 μg/mL propidium iodide (PI) staining solution to cell pellet and mixed well. Added 50 μL of RNaseA stock solution (10 mg/mL) and incubate for 30 min at room temperature. Samples were stored at 4°C until analyzed by flow cytometry (Becton-Dickinson, San Jose, CA).

### Annexin V Apoptosis Detection

The annexin V-FITC Apoptosis Detection Kit was used for flow cytometry experiment to detect apoptotic cells. VSMCs cultured in DMEM with test compounds were washed twice with cold PBS and then resuspended in 1x binding buffer at the concentration of 1 x 10^6^ cells/mL. The cell suspension was transferred to a 5-ml culture tube and mixed with 5 μL of annexin V-FITC and 10 μL of PI. The cells were gently vortexed and incubated for 15 min at room temperature in the dark. Then 400 μL of 1x binding buffer was added to each tube and analyzed by flow cytometry with the use of a FACSCalibur flow cytometer (Becton-Dickinson, San Jose, CA).

### Western blot analysis

Equal amounts of proteins (30 μg per lane) were subjected to 10% SDS-PAGE, transferred to nitrocellulose membranes (Amersham). The membranes were blocked with 5% fat-free milk in PBS containing 0.1% Tween 20 (PBST) for 1 h and followed by immunoblotting with antibodies for nuclear factor (NF)-κB, IκBα, phospho-p38 mitogen-activated protein kinase (MAPK), p38 MAPK, bcl-2, C23, or α-tubulin (Santa Cruz Biochemicals). Subsequently, membranes were washed three times with PBST, incubated with secondary horseradish peroxidase (HRP)-conjugated antibodies (Santa Cruz Biochemicals), and again followed by three washes. The signals were then visualized with an enhanced chemiluminescence detection system (Amersham). Exposures were recorded on X-film (Fuji).

### IL-6 and NO assays

Cells (2 x 10^5^ cells/mL) were serum-starved for 48 h and then treated with test compounds for 24 h. The medium was collected and centrifuged at 500 rpm for 1 min. The supernatant was stored at -70°C until assay. ELISA for rat IL-6 was performed with an ELISA kit (Pierce Endogene) according to the manufacturer’s instructions. Moreover, the NO (nitrite/nitrate) levels were determined using the nitrite/nitrate colorimetric assay kit (R&D Systems).

### Statistical analysis

Data are expressed as mean ± SEM of a variable number of experiments or displayed as representative observations of at least three separate experiments. Statistical significance was assessed by one way analysis of variance (ANOVA) and Dunnett’s test. The significant difference is determined when p-value is less than 0.05.

## Results

### Benzo[a]pyrene suppressed NO-induced death and apoptosis in VSMCs

There was no change on total cell number between control and benzo[a]pyrene (1–30 μmol/L)-treated VSMCs under serum deprivation culture condition (S1A Fig). We next observed the cell cycle distribution in VSMCs under serum-free condition, and confirmed that no change between control and benzo[a]pyrene (10 μmol/L)-treated VSMCs (S1B Fig). These results indicated that benzo[a]pyrene did not cause cell death of VSMCs in serum-free condition.

**Fig 1:**
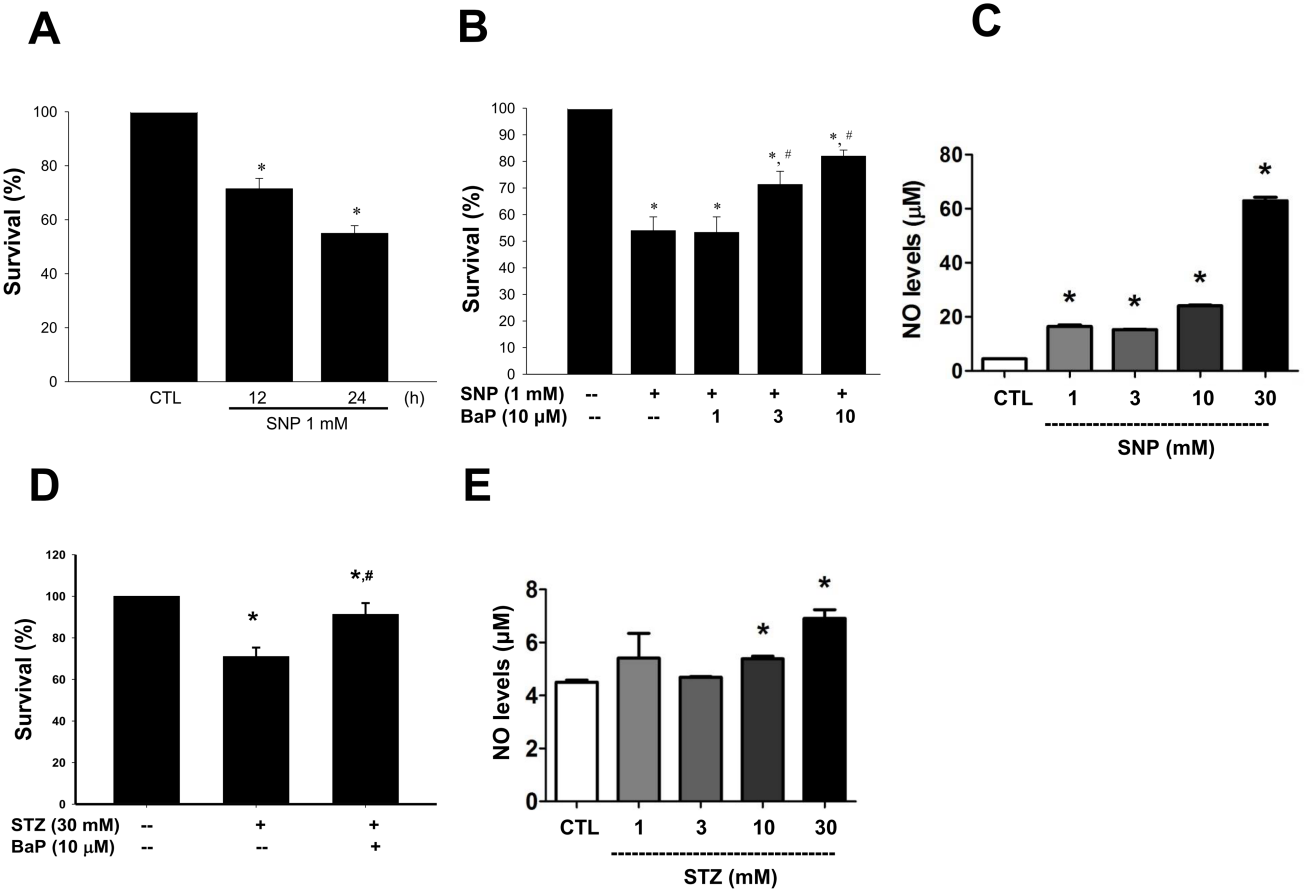
Effect of NO donors on cell viability and NO release in the presence or absence of benzo[a]pyrene in VSMCs. (A) VSMCs were cultured in serum-free DMEM in the presence or absence of sodium nitroprusside (SNP, 1 mmol/L) for 12 or 24 h. (B) VSMCs were treated with SNP (1 mmol/L) in the presence or absence of benzo[a]pyrene (10 μmol/L) for 24 h. (C) VSMCs were treated with SNP (1–30 mmol/L) for 24 h. (D) VSMCs were treated with streptozotocin (STZ, 30 mmol/L) in the presence or absence of benzo[a]pyrene (10 μmol/L) for 24 h. (E) VSMCs were treated with streptozotocin (STZ, 1–30 mmol/L) for 24 h. Cell viability was determined by MTT assay. Cell survival was expressed as % of untreated control. The NO (nitrite/nitrate) levels were determined using the nitrite/nitrate colorimetric assay kit. All data are represented as mean ± SEM from three independent experiments. *P < 0.05 as compared with the control. #*P* < 0.05 as compared with SNP alone (B) or STZ alone (D).

In quiescent VSMCs, NO donor SNP (1 mmol/L) time-dependently suppressed cell viability by 28.5% (12 h) and 46% (24 h), respectively (Fig 1A). Co-incubation with benzo[a]pyrene (1–10 μmol/L) for 24 h dose-dependently reversed the inhibition of cell viability induced by SNP (Fig 1B). SNP (1–30 mmol/L) effectively increased the NO release in a dose-dependent manner (Fig 1C). Moreover, we also used another NO donor- streptozotocin [32], which is a glucosamine-nitrosourea compound, to confirm the effect of benzo[a]pyrene on VSMCs viability. As shown in Fig 1D, streptozotocin (30 mmol/L) effectively decreased the VSMCs viability, which could be significantly reversed by benzo[a]pyrene (10 μmol/L). Streptozotocin (1–30 mmol/L) could increase the NO release in a dose-dependent manner (Fig 1E).

**Fig 2:**
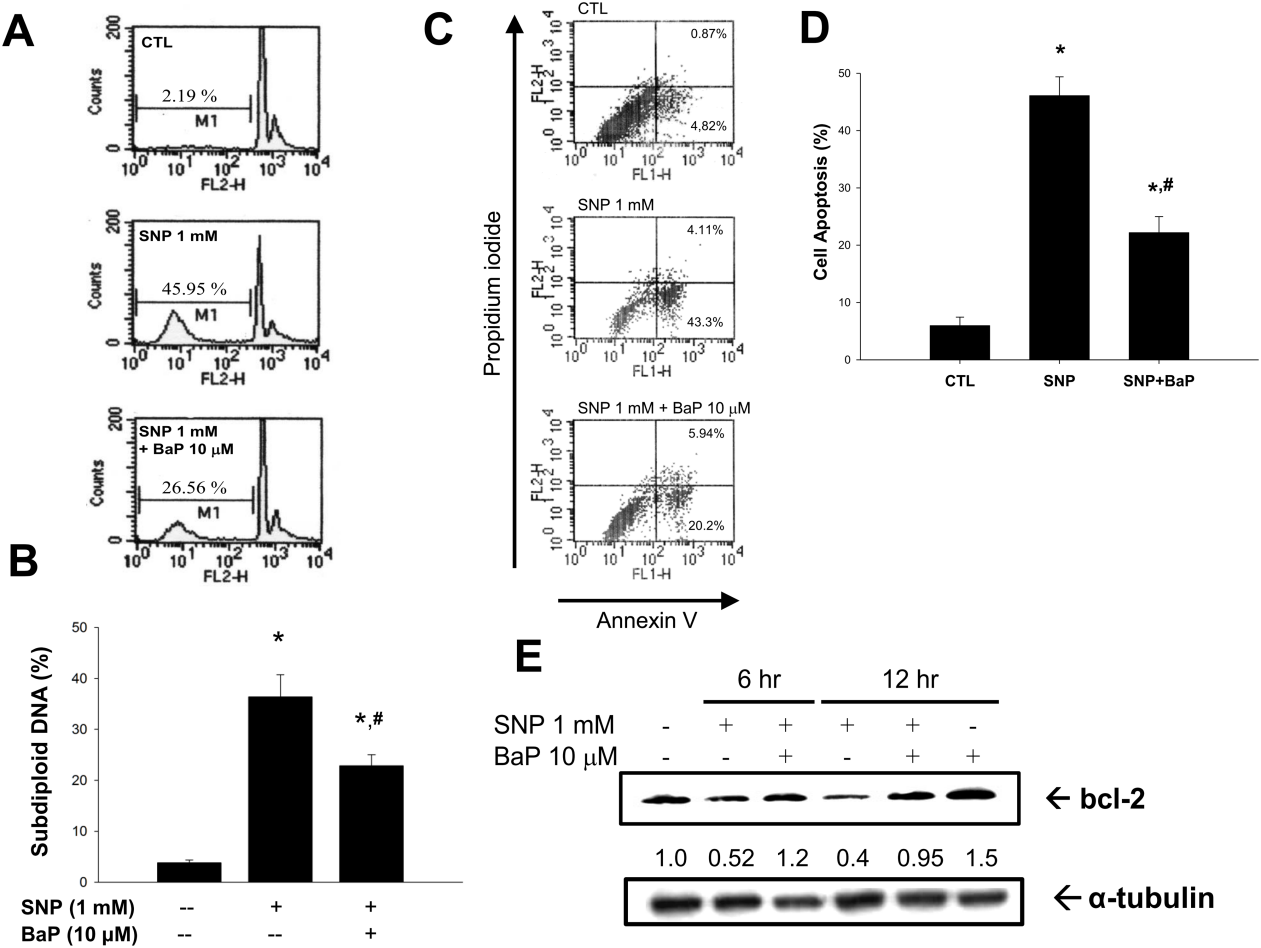
Effect of NO donor on subdiploid DNA content and cell apoptosis in the presence or absence of benzo[a]pyrene in VSMCs. VSMCs were cultured in serum-free DMEM. VSMCs were treated with SNP (1 mmol/L) in the presence or absence of benzo[a]pyrene (10 μmol/L) for 24 h (A and B) or 12 h (C and D). (A) and (B) Cells were fixed with 70% ethanol and stained with propidium iodide (PI), and the DNA content was analyzed by flow cytometry. Representative images are shown (A). The percentage of subdiploid DNA content in cells was calculated (B). (C) and (D) The cells were collected, and stained with Annexin V-FITC and PI. The percentage of annexin V-positive (annexin V (+)), PI-negative (PI (-)) or PI (+) cells was calculated from fluorescence-1 (FL1-H) / fluorescence-2 (FL-2-H) dot plots, and is shown in the respective upper right and lower right hand corner. Data are represented as mean ± SEM from six independent experiments. *P < 0.05 as compared with control. #*P* < 0.05 as compared with SNP alone. (E) VSMCs were treated with SNP (1 mmol/L) in the presence or absence of benzo[a]pyrene (10 μmol/L) for 6 or 12 h. The protein expression of bcl-2 and α-tubulin (internal control) was determined by Western blotting. One representative experiment of three is shown.

We next analyzed the subdiploid DNA population in VSMCs by flow cytometry. The subdiploid DNA content was markedly increased after SNP stimulation by 36% (Fig 2A and 2B). Benzo[a]pyrene (10 μmol/L) treatment significantly reversed SNP-increased subdiploid DNA levels (Fig 2A and 2B). The annexin V-FITC and PI staining was further used to analyze the percentage of apoptotic cells. As shown in Fig 2C and 2D, the late apoptotic cells and early apoptotic cells were increased from 0.9% to 4.1% or 4.8% to 43.3%, respectively, when cells were treated with SNP for 12 h. Total percentage of apoptotic cells was increased from 5.7% to 47.4%. Once benzo[a]pyrene (10 μmol/L) was co-incubated with SNP, total percentage of apoptotic cells shifted to 26.1% (Fig 2C and 2D). Moreover, SNP markedly decreased the protein expression of bcl-2 in VSMCs, which could be effectively reversed by benzo[a]pyrene (Fig 2E). These results indicated that benzo[a]pyrene was capable of inhibiting SNP-induced apoptosis of VSMCs.

### Role of IL-6 in anti-apoptotic effect of benzo[a]pyrene

**Fig 3:**
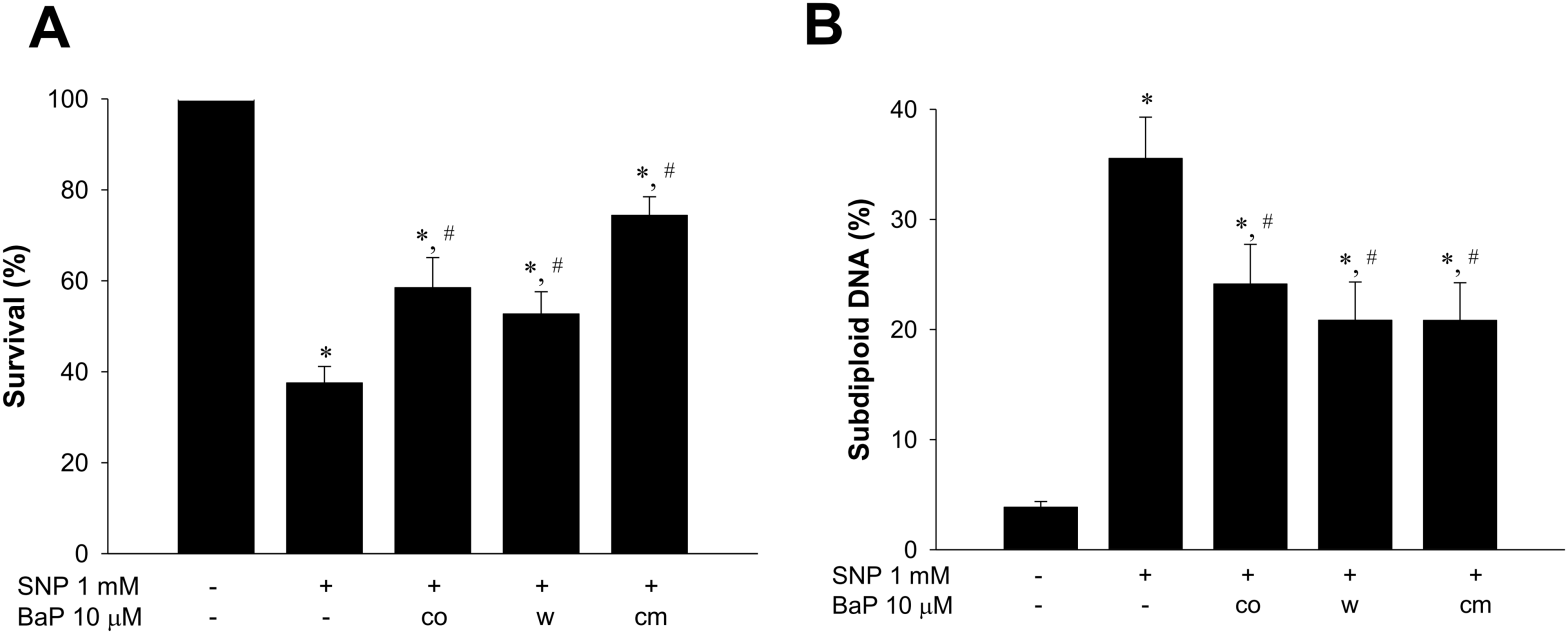
Inhibition of cell viability by NO donor was partially suppressed by conditioned medium of benzo[a]pyrene-treated cells. VSMCs were cultured in serum-free DMEM in the presence or absence of benzo[a]pyrene (10 μmol/L) for 24 h and then the condition medium was collected. Cells were refreshed with DMEM containing SNP (1 mmol/L) as “w”. The condition medium was added to another cell culture dish as “cm”. Cells were co-incubated with SNP and benzo[a]pyrene as “co”. After 24 h, the cell viability was determined by MTT assay (A) and the subdiploid DNA content was determined by flow cytometry (B). Data are represented as mean ± SEM from three independent experiments. *P < 0.05 as compared with control. #*P* < 0.05 as compared with SNP alone.

**Fig 4:**
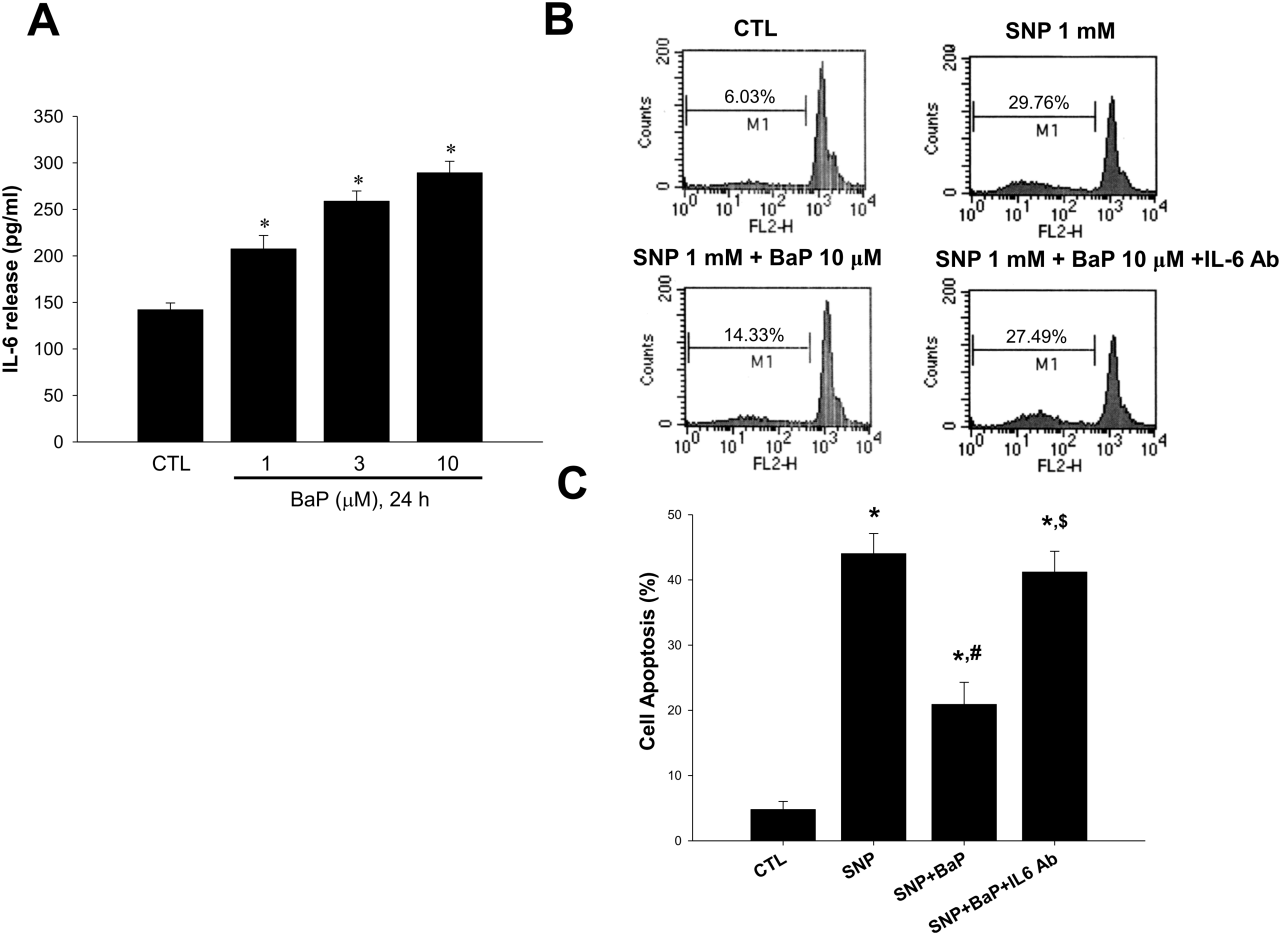
Role of interleukin-6 (IL-6) in the anti-apoptotic effect of benzo[a]pyrene on SNP-treated VSMCs. (A) VSMCs were cultured in serum-free DMEM in the presence or absence of benzo[a]pyrene for 24 h. The IL-6 production was measured by ELISA. Data are represented as mean ± SEM from three independent experiments. *P < 0.05 as compared with the control. (B) VSMCs were treated with SNP (1 mmol/L) in the presence or absence of benzo[a]pyrene (10 μmol/L) for 24 h. The subdiploid DNA content was determined by flow cytometry. Representative images are shown. (C) VSMCs were treated with SNP (1 mmol/L) in the presence or absence of benzo[a]pyrene (10 μmol/L) for 12 h. The annexin V-FITC and PI staining was analyzed by flow cytometry. Data are represented as mean ± SEM from three independent experiments. #*P* < 0.05 as compared with SNP alone. $*P* < 0.05 as compared with SNP+benzo[a]pyrene.

To study the signaling involved in the anti-apoptotic effect of benzo[a]pyrene, we collected conditioned media (cm) from cells treated with benzo[a]pyrene for 24 h and then added it to another cultured cells following treatment with SNP. The results showed that benzo[a]pyrene-condition media enabled to prevent decreased cell viability (Fig 3A) and increased subdiploid DNA content (Fig 3B) in VSMCs by SNP challenge. Besides, both decreased cell viability (Fig 3A) and increased subdiploid DNA content (Fig 3B) were also attenuated during the condition in which cells treated with benzo[a]pyrene for 24 h were then washed and changed to fresh culture media containing SNP alone (w). These results indicated that some mediators might be induced and secreted to media by which benzo[a]pyrene prevented cell death in an autocrine manner. Since IL-6 has been reported to prevent apoptosis in various cell types [33,34], we next investigated if benzo[a]pyrene was able to stimulate IL-6 release. As shown in Fig 4A, benzo[a]pyrene does-dependently increased the production of IL-6 in VSMCs. We next investigated the involvement of IL-6 in anti-apoptotic effect of benzo[a]pyrene. Blockade of IL-6 with the neutralizing antibody (2 μg/mL) abolished benzo[a]pyrene-reduced subdiploid DNA content and apoptosis in SNP-treated VSMCs (Fig 4B and 4C). These results showed that IL-6 produced by VSMCs contributed to anti-apoptotic effect of benzo[a]pyrene on NO-related VSMCs apoptosis.

### Involvement of NF-κB and p38 MAPK in the anti-apoptotic effect of benzo[a]pyrene

**Fig 5:**
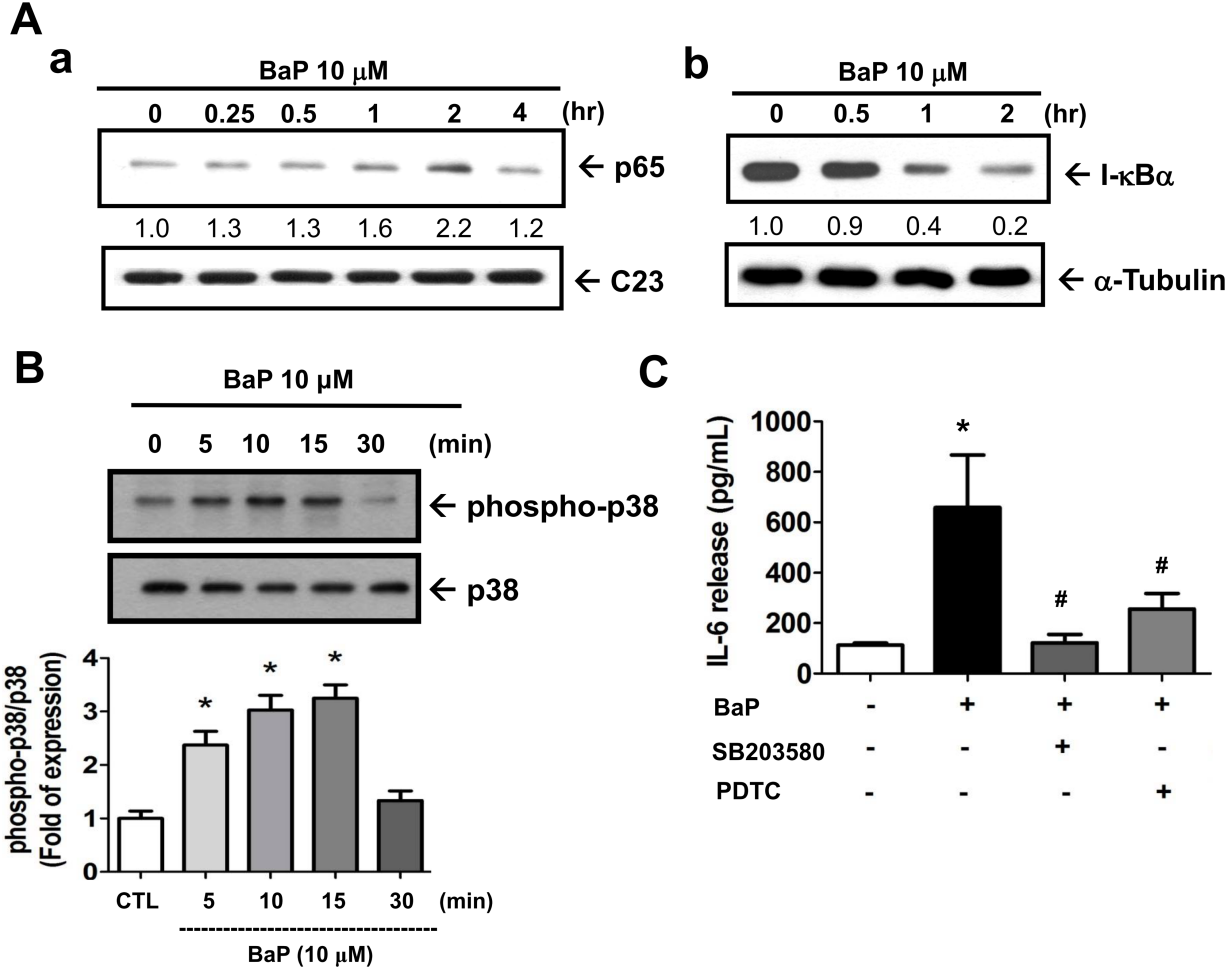
Benzo[a]pyrene induced the activation of both NF-κB and p38 MAPK, which were involved in the benzo[a]pyrene-increased IL-6 production in VSMCs. VSMCs were cultured in serum-free DMEM in the presence or absence of benzo[a]pyrene (10 μmol/L) for 0.25–4 h (A) or 5–30 min (B). The protein expressions of NF-κB-p65 (nuclear protein) and IκBα (A) and phospho-p38/p38 MAPK (B) were determined by Western blotting. The protein fold changes, which were normalized to C23, α-tubulin, or p38, were shown below each blot. Experiments were repeated three times. One representative experiment is shown. (C) VSMCs were cultured in serum-free DMEM in the presence or absence of benzo[a]pyrene for 24 h. The IL-6 production was measured by ELISA. Data are represented as mean ± SEM from three independent experiments. *P < 0.05 as compared with control. #*P* < 0.05 as compared with benzo[a]pyrene alone.

**Fig 6:**
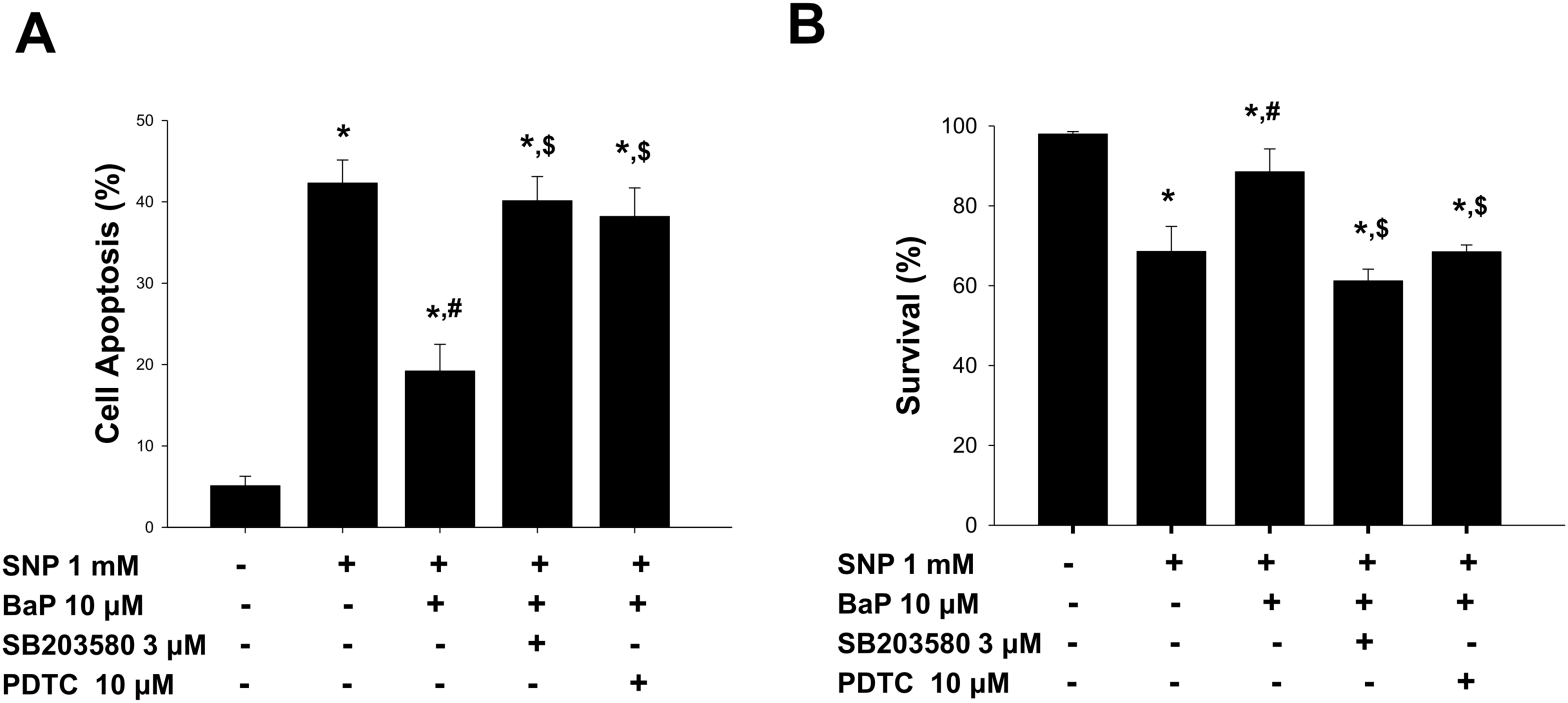
Role of NF-κB and p38 MAPK in the anti-apoptotic effect of benzo[a]pyrene on SNP-treated VSMCs. VSMCs were pretreated with SB203580 (3 μmol/L) or PDTC (10 μmol/L) followed by treatments of benzo[a]pyrene (10 μmol/L) and SNP (1 mmol/L) for 12 h. The annexin V-FITC and PI staining was analyzed by flow cytometry (A). Cell viability was determined by MTT assay (B). Data are represented as mean ± SEM from three independent experiments. *P < 0.05 as compared with the control. #*P* < 0.05 as compared with SNP alone. $*P* < 0.05 as compared with SNP+benzo[a]pyrene.

Both NF-κB and p38 MAPK signals possess the ability to regulate VSMCs proliferation [35,36] and IL-6 induction [37]. To investigate the signaling involved in the anti-apoptotic effect of benzo[a]pyrene in VSMCs, we tested whether NF-κB and p38 MAPK were involved. As shown in Fig 5A-a, the nuclear NF-κB-p65 protein expression in VSMCs was time-dependently increased by benzo[a]pyrene (10 μmol/L). The protein expression of IκBα was time-dependently decreased by benzo[a]pyrene (10 μmol/L) (Fig 5A-b). The phosphorylation of p38 MAPK in VSMCs was also time-dependently increased by benzo[a]pyrene (10 μmol/L) (Fig 5B). Moreover, both NF-κB inhibitor PDTC (10 μmol/L) and p38 MAPK inhibitor SB203589 (3 μmol/L) significantly suppressed the benzo[a]pyrene-increased IL-6 production in VSMCs (Fig 5C). Both PDTC (10 μmol/L) and SB203589 (3 μmol/L) could also significantly inhibit the anti-apoptotic effect (Fig 6A) and survival enhancement (Fig 6B) of benzo[a]pyrene against SNP challenge. These results implicated that benzo[a]pyrene inhibited SNP-induced VSMCs apoptosis through the activation of NF-κB and p38 MAPK signals.

## Discussion

The present study provides the first characterization of the effect of benzo[a]pyrene on the regulation of apoptosis in VSMCs. Our observations also suggest that the survival signal by benzo[a]pyrene is mediated from IL-6 release because the neutralizing antibody to IL-6 inhibits benzo[a]pyrene-induced anti-apoptotic effect.

Low-concentration NO is considered to regulate the physiological functions, but high-concentration NO may contribute to the pathological effects [13]. The physiological NO levels appear to be in the range from 1 μmol/L to 10 nmol/L with the short half-life (9 to 900 min) [38]. The range of NO levels in bloods of human or mammalian has been estimated to be 3 nmol/L up to 20 μmol/L [39]. The exhaled NO concentrations in acute asthma children were significantly higher (31.3 ± 4.2 ppb (μg/L)) than in healthy children (5.4 ± 0.4 ppb (μg/L)) [40]. The serum total concentrations of NO (NO_3_^−^/NO_2_^−^) in control subjects and squamous cell carcinoma of the oral cavity patients in IV stage were about 12 and 30 μmol/L, respectively [41]. In the present study, the NO levels in culture medium of control VSMCs and SNP (1 mmol/L)-treated VSMCs were about 4.5 and 15 μmol/L, respectively. Therefore, we used a NO donor at the released NO concentrations relevant to human exposure in bloods to test its cytotoxicity to VSMCs.

Apoptosis is known to as a physiological suicide pathway to maintain the homeostasis of tissue organs. VSMCs are major constituents of the medial layer of blood vessels and are involved in the development of atherosclerotic plaque by abnormal accumulation in intimal vessels [2,3]. NO-induced VSMCs apoptosis may be an important determinant to regulate cell number of normal arterial wall and is a feature of atherosclerosis pathology [13,18]. The complex mechanisms of NO-mediated apoptosis have been mentioned. NO donor has been shown to potentiate DNA damage and alter DNA repair in ionizing radiation-treated cells [42]. NO could also inhibit the catalytic activity of the 26S proteasome and regulate proteasomal subunit expression in VSMCs [15]. An increased susceptibility to NO-induced VSMC apoptosis has been observed in p53(-/-) cells, which could be effectively abrogated by antioxidant catalase [43]. Besides, the protein expression of anti-apoptotic protein was decreased under SNP exposure in VSMCs [44]. In the present study, we also found that SNP induced bcl-2 degradation, apoptosis, and cell death in primary rat VSMCs, which could be significantly reversed by benzo[a]pyrene. These results suggest that benzo[a]pyrene is capable of suppressing NO-induced apoptosis and cell death in VSMCs.

Atherosclerosis has been suggested to be an inflammatory disease [45,46]. A significant role of IL-6 in the pathophysiology of atherosclerosis has also been suggested [24,45]. VSMCs secrete copious IL-6 under stimulation conditions such as tumor necrosis factor (TNF)-α [47], IL-1β [48], platelet-derived thrombin [49], endothelin I [50], and lipopolysaccharide [37] that they may be involved in the pathogenesis of atherosclerosis. There are several important regulatory cis DNA elements in the promoter region of the IL-6 gene such as AP-1, CRE, NF-IL6, and NF-κB, which are conserved among species such as mice, rat and human, and regulate IL-6 gene expression in a cell-specific manner [51,52]. Recombinant interleukin-6 administration has been found to protect MIN6 β-cells from NO dependent cytokine-induced apoptosis and reduced bcl-2/bax protein ratio [53]. The pleiotropic action of IL-6 has also been found to improve the NO-induced cytotoxic CD8+ T cell dysfunction from chagasic patients [54]. IL-6 was capable of inducing bcl-2 expression to protecting cell functions in response to hyperoxia toxicity in human umbilical vein endothelial cells (HUVECs) [55]. It has been demonstrated that NO triggers cell death by regulating anti-apoptotic bcl-2 family members in mouse embryonic fibroblasts [56]. In the present study, we found that benzo[a]pyrene dose-dependently and significantly increased the IL-6 production and inhibited the reduced bcl-2 expression in SNP-treated VSMCs. IL-6 neutralizing antibody could significantly reverse the anti-apoptotic effect of benzo[a]pyrene on SNP-treated VSMCs. These findings suggest that IL-6 plays an important role in the atherogenic effect of benzo[a]pyrene.

The activation of NF-κB has been shown to play an important role in angiotensin II-dependent VSMCs proliferation [35]. Benzo[a]pyrene has been shown to induce rapid NF-κB activation via redox regulation in VSMCs [57,58]. Mehrhof et al. (2005) have suggested that NF-κB signaling may be as a regional regulator of VSMCs survival rather than a direct promoter of VSMCs proliferation [58]. Moreover, p38 MAPK signaling has also been demonstrated to be involved in the serum-induced VSMCs proliferation [36]. A p38 MAPK-dependent signaling pathway has been found to contribute to the perivascular adipose tissue-derived leptin-triggered VSMCs phenotypic switching [59]. On the other hand, transcriptional activation of cytokine genes commonly requires the induction of NF-κB [60]. It has been reported that pretreatment of human airway smooth muscle cells with p38 MAPK inhibitor SB203580 significantly inhibited the secretion of IL-6 after TNF-αstimulation [41]. TNF-α has also been found to induce p38-dependent IL-6 induction and protect cardiac myocytes from apoptosis [61]. The angiotensin II-induced IL-6 gene expression also depends on NF-κB activation in VSMCs [62]. Both NF-κB and p38 MAPK signals have also been shown to be involved in the lipopolysaccharide-induced IL-6 induction in VSMCs [37]. The intracellular signaling pathways by which benzo[a]pyrene leads to cell survival and IL-6 production in SNP-treated VSMCs are of interest. In the present study, we confirmed that benzo[a]pyrene in deed activated NF-κB to translocate to nucleus, and PDTC, an inhibitor of NF-κB activation, abolished the anti-apoptotic effect of benzo[a]pyrene. We also found that benzo[a]pyrene markedly increased the phosphorylation of p38 MAPK in VSMCs. SB203580 could also inhibit the benzo[a]pyrene-induced anti-apoptotic effect, suggesting that p38 MAPK signaling pathway is involved in the benzo[a]pyrene-induced anti-apoptotic effect. We further found that both NF-κB and p38 MAPK inhibitors significantly inhibit the benzo[a]pyrene-induced IL-6 production. The protein kinase C (PKC)-related signaling has also been shown to be involved in the IL-6 production induced by serotonin from human VSMCs [63]. However, Funakoshi et al reported that angiotensin II-induced IL-6 expression was dependent on intracellular Ca^2+^, tyrosine phosphorylation, and ERK activation, and independent of PKC and extracellular Ca^2+^ [64]. These findings suggest that regulation of IL-6 may be complex and needs more studies to understand the mechanisms by which benzo[a]pyrene induces IL-6 release. On the other hand, benzo[a]pyrene has been found to inhibit angiogenesis in HUVECs via an aryl hydrocarbon receptor (AhR)-dependent pathway [65]. The coplanar polychlorinated biphenyls (PCBs), the AhR agonists, can disrupt endothelial barrier function and promote IL-6 production in porcine endothelial cells; but PCB 153, which is not a ligand for the AhR, had no effect on endothelial function and IL-6 production [66]. Hu et al. recently showed that BaP induced IL-6 production and inhibited sebum production in human sebocytes via the activation of AhR signaling [30]. Therefore, in this study, benzo[a]pyrene induced IL-6 production in VSMCs may through an AhR signaling pathway.

**Fig 7:**
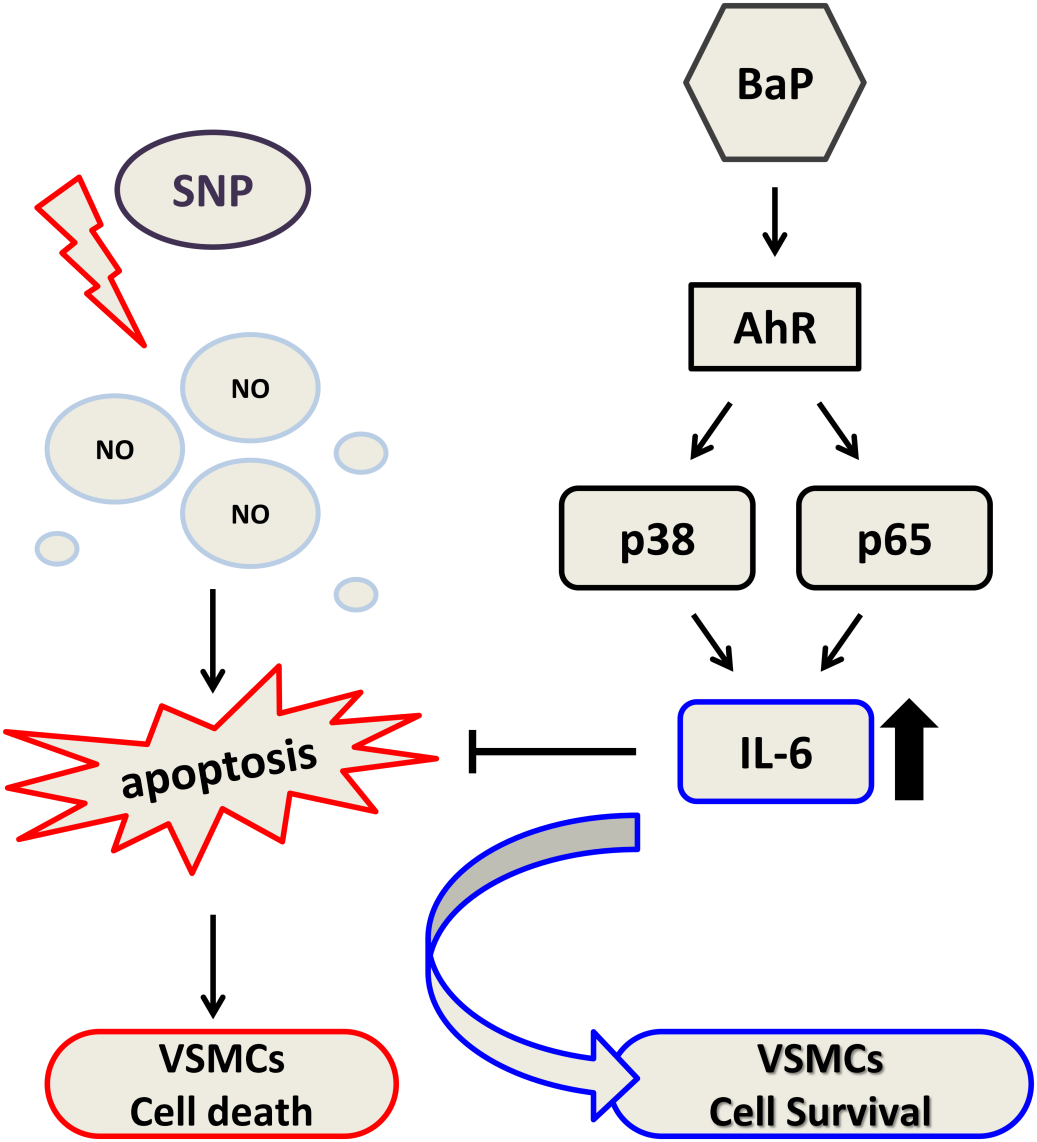
A proposed model showed the ability of benzo[a]pyrene to suppress a death signal in VSMCs triggered by NO through an IL-6 signaling pathway.

Indeed, not only proliferation of VSMCs but also apoptosis is found in atherosclerotic lesions [2,18], suggesting that apoptosis may be a compensatory behavior to repair vascular injury. Dysfunction of the apoptosis process has been linked to pathogenesis of cancer and atherosclerosis [67,68]. The present study showed the ability of benzo[a]pyrene to suppress a death signal in VSMCs triggered by NO through an IL-6 signaling pathway (Fig 7). These findings propose a new mechanism for the atherogenic effect of benzo[a]pyrene. Benzo[a]pyrene may therefore not only alter VSMCs to a proliferative phenotype, but also exert an anti-apoptotic effect participating in vascular disease. It is conceivable that the ability of benzo[a]pyrene to inhibit NO-induced cell death may play a substantial role in atherosclerotic lesion formation. Further studies are necessary to define the anti-apoptotic effect of benzo[a]pyrene on the pathogenesis of vascular lesion *in vivo*.

## Supporting information

S1 Fig Effects of benzo[a]pyrene on cell number and cell cycle distribution in VSMCs. (A) VSMCs were cultured in serum-free DMEM in the presence or absence of benzo[a]pyrene (1–30 μmol/L). After 72 h, cells were collected, stained with trypan blue, and counted by hemocytometry. Data are presented as mean ± SEM from three independent experiments. (B) VSMCs were cultured in serum-free DMEM in the presence or absence of benzo[a]pyrene (10 μmol/L) for 72 h. The DNA content was analyzed by flow cytometry. One representative experiment of three is shown.
(TIF)
