# Supplementary figures and images for: Benzo[a]pyrene activates interleukin-6 induction and suppresses nitric oxide-induced apoptosis in rat vascular smooth muscle cells
Source: PLoS One. 2017 May 22;12(5):e0178063. doi: 10.1371/journal.pone.0178063 (PMC5439712; doi:10.1371/journal.pone.0178063)

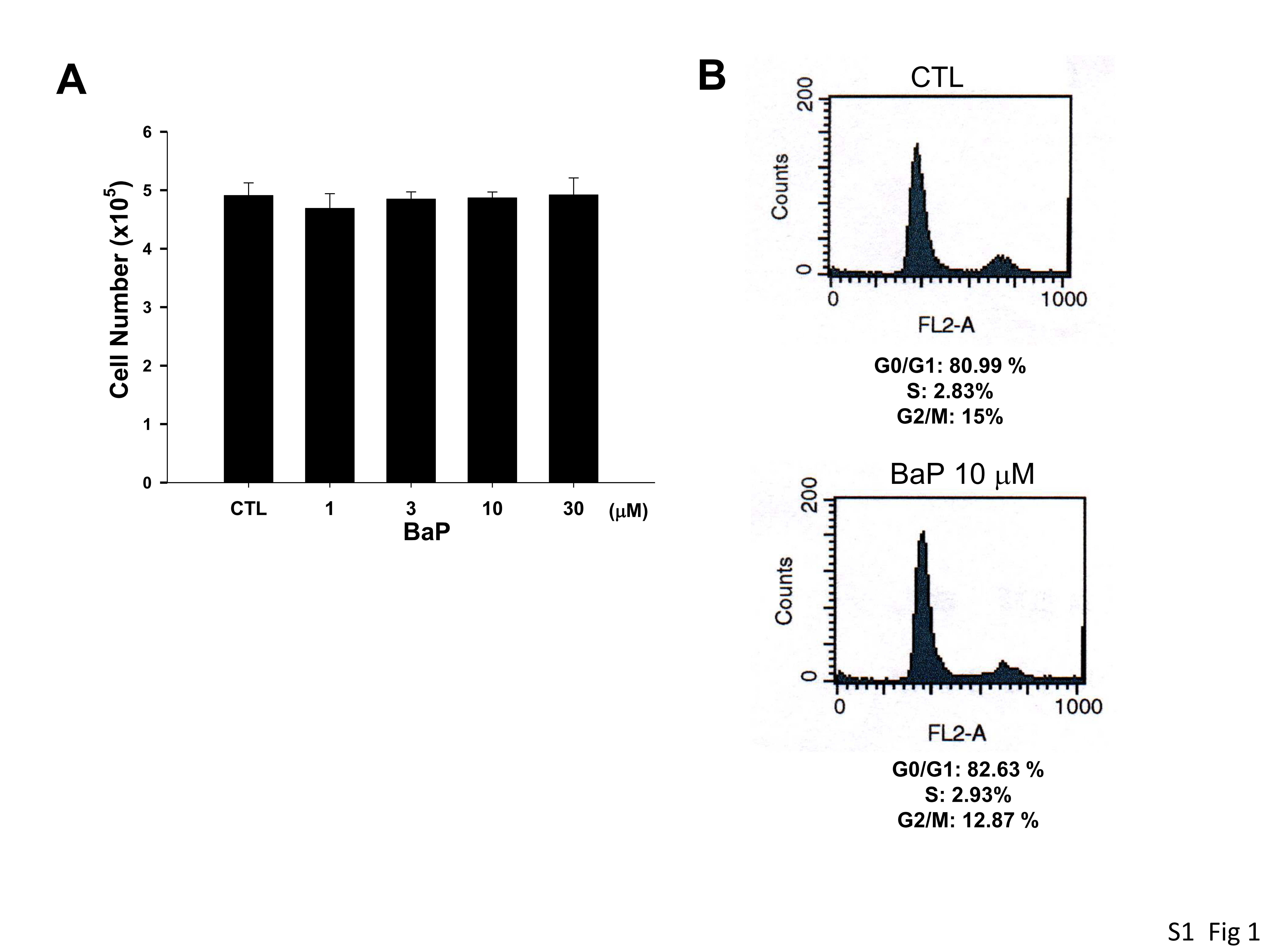

Supplement: S1 Fig — (A) VSMCs were cultured in serum-free DMEM in the presence or absence of benzo[a]pyrene (1–30 μmol/L). After 72 h, cells were collected, stained with trypan blue, and counted by hemocytometry. Data are presented as mean ± SEM from three independent experiments. (B) VSMCs were cultured in serum-free DMEM in the presence or absence of benzo[a]pyrene (10 μmol/L) for 72 h. The DNA content was analyzed by flow cytometry. One representative experiment of three is shown. (TIF) [file pone.0178063.s001.tif]
